# Efficacy of a single-dose topical formulation combining Fipronil, Moxidectin and Praziquantel against natural infestations of *Notoedres cati*, *Otodectes cynotis* and *Felicola subrostratus* in cats

**DOI:** 10.1590/S1984-29612025080

**Published:** 2026-04-10

**Authors:** Diefrey Ribeiro Campos, Eduardo Fellipe Melo Santos Soares, Breno Cayeiro Cruz, Gabriela Pereira Salça de Almeida, Gessica Ariane de Melo Cruz, Milenni Garcia Michels, Ferdinando Nielsen de Almeida, Igor Renan Honorato Gatto, Thais Ribeiro Correia, Fabio Barbour Scott

**Affiliations:** 1 Universidade Federal Rural Rio de Janeiro, Instituto de Medicina Veterinária, Departamento de Parasitologia Animal, Laboratório de Quimioterapia Experimental em Parasitologia Veterinária - LQEPV, Seropédica, RJ, Brasil; 2 Ourofino Saúde Animal Ltda, Cravinhos, SP, Brasil

**Keywords:** Treatment, feline, parasites, mange, lice, Tratamento, felino, parasitos, sarna, piolhos

## Abstract

Lice and mange represent common ectoparasitic infestations in domestic cats, affecting their health and well-being. The study evaluated the efficacy of a topical formulation containing 12.5% fipronil + 0.83% moxidectin + 8.3% praziquantel against common feline ectoparasites *Otodectes cynotis, Notoedres cati* and *Felicola subrostratus*. For the ear mite study, a controlled trial was conducted on cats naturally infested with *O. cynotis*. For the other two parasites, field studies were conducted on naturally infested cats. Cats in the treated group received a single topical dose of formulation. Parasite counts were performed at various time points post-treatment to assess efficacy. The results demonstrated the formulation was highly effective against all three parasites, achieving 100% efficacy after 3 days of treatment. The product was well-tolerated and safe, making it a suitable option for the treatment of feline ectoparasitosis.

## Resumo

Piolhos e ácaros causadores de sarnas representam infestações de ectoparasitos comuns em gatos domésticos, afetando sua saúde e bem-estar. O estudo avaliou a eficácia de uma formulação tópica contendo 12,5% de fipronil + 0,83% de moxidectina + 8,3% de praziquantel contra ectoparasitos felinos comuns *Otodectes cynotis*, *Notoedres cati* e *Felicola subrostratus*. Para o estudo do ácaro de ouvido, um ensaio controlado foi conduzido em gatos naturalmente infestados com *O. cynotis*. Para os outros dois parasitos, estudos a campo foram conduzidos em gatos naturalmente infestados. Gatos do grupo tratado receberam uma única dose tópica da formulação. Contagens de parasitos foram realizadas em vários momentos pós-tratamento para avaliar a eficácia. Os resultados demonstraram que a formulação foi altamente eficaz contra todos os três parasitos, alcançando 100% de eficácia 3 dias após o tratamento. O produto foi tolerado e seguro, tornando-o uma opção adequada para o tratamento de ectoparasitoses felinas.

## Introduction

Lice and mange are common ectoparasitic infestations in domestic cats, adversely affecting their health and well-being (Miller et al., 2012). Among the principal causes of scabies,*Notoedres cati*and*Otodectes cynotis*are frequently identified in domestic cats (Premaalatha et al. 2023; Rataj et al. 2004; Thomas et al. 2016). *Notoedres cati* is highly contagious and primarily transmitted through direct contact, with occasional zoonotic implications that may result in transient cutaneous disease in humans (Santos et al., 2019). *Otodectes cynotis*accounts for fewer than half of feline otitis externa cases (Campos et al., 2019). The louse*Felicola subrostratus*, commonly referred to as the cat louse, is a species-specific ectoparasite of felines (Figueiredo et al., 2013). Infestations by these parasites can cause intense pruritus, skin irritation, and secondary infections if left untreated, thereby significantly compromising feline welfare (Miller et al., 2012).

In veterinary practice, parasite control remains a central focus due to rising drug resistance and evolving client expectations, which necessitate the continual development of new antiparasitic agents. The use of combination therapies is often favored in the development of novel treatments, as these formulations broaden the antiparasitic spectrum and reduce the number of required applications (Arisov et al., 2018).

Furthermore, administering medications to cats presents practical challenges for owners, particularly due to aversions to the taste of oral formulations and the high incidence of non-compliance. This often results in behavioral resistance during administration (Sivén et al., 2017). In this context, topical treatments offer a promising alternative.

The novel topical formulation combines fipronil, moxidectin, and praziquantel. While fipronil, a phenylpirazole, is a contact insecticide and acaricide widely recognized for its efficacy against ectoparasites (Gant et al., 1998), moxidectin, a macrocyclic lactone, acts against a variety of nematodes and ectoparasites (Schraven et al., 2021). Praziquantel, in turn, is a potent broad-spectrum anthelmintic, effective against numerous species of cestodes and trematodes (Chai, 2013). This combined approach simplifies antiparasitic management, reducing stress for the animal and ensuring more comprehensive protection against multiple infestations.

This study aimed to evaluate the efficacy of a topical formulation containing 12.5% fipronil, 0.83% moxidectin and 8.3% praziquantel administered at the recommended dosage for treating cats naturally infested with *N. cati*, *O. cynotis*, and*F. subrostratus*.

## Material and Methods

### Study design

All three studies followed a controlled, randomized, and investigator-blinded protocol and were conducted in the state of Rio de Janeiro, in the Southeast region of Brazil. Each study included a treated group, which received the investigational product (Banni^3^; 12.5% fipronil, 0.83% moxidectin, 8.3% praziquantel; Ourofino Saúde Animal Ltda.), and one or more control groups (positive, negative, or both). The studies were performed either under field conditions or at the *Laboratório de Quimioterapia Experimental em Parasitologia Veterinária* (LQEPV), Veterinary Institute, *Universidade Federal Rural do Rio de Janeiro*, and involved a total of 47 cats naturally infested with ectoparasites. Enrolled cats were either male or non-pregnant females, aged between 5 and 190 months, and weighed between 0.8 and 5.1 kg. None had received antiparasitic treatment in the two months preceding the study. All procedures adhered to the AAFP/ISFM and VICH Guideline GL9 for Good Clinical Practice (Taylor et al., 2022; European Agency for the Evaluation of Medicinal Products, 2000). For the field studies, informed consent was obtained from the owners on day −7 prior to any procedure. During field studies, all animals remained under the care of their respective owners. Upon controlled test completion, cats enrolled in the controlled study were returned to their original animal facility and collective cattery. Daily health checks were conducted to ensure that all animals remained healthy throughout the study period. Detailed veterinary examinations were performed on the day of enrollment and at study completion.

### Treatment

Treatment was administered topically on day 0 to the dorsal region of each cat in the treated groups, based on individual weight: 0.3 mL for animals ≤2.5 kg and 0.9 mL for those between 2.6 and 7.5 kg. In the*O. cynotis*study, a positive control group received 6% selamectin at the recommended dose (0.25 mL for ≤2.5 kg; 0.75 mL for 2.6–7.5 kg). For the *N. cati*study, a positive control group received a combination of 10% imidacloprid and 1% moxidectin (0.4 mL for ≤4.0 kg; 0.8 mL for 4.1–78.0 kg). Negative control groups in the*O. cynotis*and*F. subrostratus*studies remained untreated.

### Efficacy against*Otodectes cynotis*

To evaluate efficacy against*O. cynotis*, 21 naturally infested cats from LQEPV were selected and randomly assigned to three groups of seven animals each. The study was conducted under controlled laboratory conditions with individualized housing, natural lighting, and species-appropriate ambient temperature. Block randomization was employed based on sex and pre-treatment mite counts. Two days prior to treatment, cats were stratified by sex and ranked in descending order of average pre-treatment mite counts. Groups were balanced for sex: the negative control group included 3 males and 4 females; the positive control (6% selamectin) group, 3 males and 4 females; and the investigational treatment group, 4 males and 3 females. Inclusion criteria required confirmation of infestation with at least five live*O. cynotis*mites in each ear canal, detected by videotoscopy on days −7 and −2 (Table 1) (Becskei et al., 2017; Taenzler et al., 2017).

**Table 1:** Number of cats naturally infested with *Otodectes cynotis*, determined by direct visualization during otoscopic examination, and corresponding mite scores in treated and untreated groups.

**Group**	**Experimental day**						
	-7	-2	3	7	14	21	28
**Negative Control Group**							
Positive animals	7	7	7	7	7	7	7
Mite score (min – max)	7-15.5	7-15.6	9.5-15	8-15.5	9-2.5	7.5-15.5	7-15
Mean (sd)	9.9 (2.9)	10.6 (2.65)	11.8 (2.3)	10.2 (2.5)	12.0 (3.1)	11.8 (2.8)	10.50 (2.6)

**Positive Control Group**							
Positive animals	7	7	0	0	0	0	0
Mite score (min – max)	8-14.5	7-13	0	0	0	0	0
Mean (sd)	10.50 (2.61)	9.9 (2.57)	0	0	0	0	0
**Efficacy (%)**	**- - -**	**- - -**	**100.0**	**100.0**	**100.0**	**100.0**	**100.0**

**Treated Group**							
Positive animals	7	7	0	0	0	0	0
Mite score (min – max)	5.5-15	7.5-19	0	0	0	0	0
Mean (sd)	10.5 (2.97)	11.4 (3.7)	0	0	0	0	0
**Efficacy (%)**	**- - -**	**- - -**	**100.0**	**100.0**	**100.0**	**100.0**	**100.0**
							
*P* value Kruskal-Wallis	0.872	<0.001	<0.001	<0.001	<0.001	<0.001	<0.001
*P* value control x positive group	-	0.000	0.000	0.000	0.000	0.000	0.000
*P* value control x treated group	-	0.000	0.000	0.000	0.000	0.000	0.000
*P* value positive x treated group	-	1.000	1.000	1.000	1.000	1.000	1.000

Negative control group = cats without treatment; Positive control = cats that have received topical 6% selamectin; Treated group = cats that have received topical administration of the investigational product; min = minimum; max = maximum; sd = standard derivation; *P* value comparing mite counts among groups.

Diagnosis was based on direct visualization of *O. cynotis* during otoscopic examination (UB CAM®). Post-treatment evaluations were conducted on days 3, 7, 14, 21, and 28 using videotoscopy. On day 28, all cats underwent ear canal lavage to confirm acaricidal efficacy (Taenzler et al., 2017). Sedation was induced using midazolam and intravenous propofol. The ear canals were filled with a 5% aqueous sodium docusate solution to soften debris, followed by aspiration using an 8-French urethral catheter and a 20 mL syringe. The aspirated material was filtered, and live mites were counted under a stereomicroscope. Selected specimens were mounted for morphological confirmation.

### Efficacy against*Notoedres cati*

The efficacy evaluation against*N. cati* involved 12 naturally infested, indoor-housed cats, all belonged to owners residing in the state of Rio de Janeiro. Six cats (4 females and 2 males) received the positive control treatment (10% imidacloprid + 1% moxidectin), while six cats (4 males and 2 females) were treated with the investigational product. Inclusion was based on the presence of cutaneous lesions characteristic of*N. cati* infestation and confirmation of live mites via skin scrapings conducted two days prior to treatment. Randomization was performed using a block design based on the order of household enrollment, with cats assigned to treatment groups in a 1:1 ratio until each group contained six animals. No negative control group was included, given the ethical concerns associated with withholding treatment for a condition that causes severe pruritus, cutaneous lesions, and a risk of secondary bacterial infections (Miller et al., 2012).

Two days before treatment, animals presenting clinical signs of notoedric mange underwent skin scrapings in affected regions. Only those with confirmed live *N. cati* mites were enrolled. Diagnosis was based on two skin scrapings (~1 cm^2^ each), followed by microscopic examination as described by Lucas (2008). Subsequent assessments occurred on days 3, 7, 14, 21, and 28 post-treatment. If no lesions were evident, scrapings were obtained from sites where infestation had previously been confirmed. Diagnostic confirmation required mite detection; following an initial negative result, two additional scrapings at weekly intervals were performed, with two consecutive negative outcomes needed to confirm resolution. In addition to mite counts, clinical signs were scored according to a standardized severity scale (Hellmann et al., 2013): Score 0: No lesions, alopecia, or pruritus; Score 1: Mild lesions, mild alopecia, occasional pruritus; Score 2: Moderate lesions, moderate alopecia, severe pruritus; Score 3: Severe lesions, extensive alopecia, crusts, intense pruritus, ulceration.

### Efficacy against*Felicola subrostratus*

The efficacy study against*F. subrostratus*was conducted as a clinical field trial in the metropolitan region of Rio de Janeiro. Fourteen naturally infested cats were enrolled, seven assigned to the negative control group (4 males, 3 females) and seven to the treatment group (3 males, 4 females). All cats were kept indoors. Inclusion criteria required detection of at least 10 live*F. subrostratus*lice during assessments conducted seven and two days before treatment, consistent with thresholds reported by Pollmeier et al. (2004). Treatment groups were formed using a block randomization method based on household enrollment, with all cats from a given household receiving the same intervention. Post-treatment evaluations occurred on days 3, 7, 14, 21, and 28.

Lice assessments were conducted at each time point across multiple anatomical regions, the head, cervical area, dorsal region (along the spine), flanks, abdomen, and tail. Each region was examined over a 5-cm span (except for the spine) by gently parting and combing the fur to count live lice without removal. The examination methodology adhered to the protocol described by Stanneck et al. (2012).

### Efficacy assessments

Efficacy (%) against*O. cynotis*and *F. subrostratus*was calculated using the following formula (Becskei et al., 2017): 


$$\left[\left(\text{C - T}\right)\text{/}\left(\text{C}\right)\right]\text{x 100}$$ (1)

where*C*is the mean number of live parasites in the negative control group at each time point, and*T*is the corresponding mean in the treated or positive control group (6% selamectin for the *O. cynotis* study).

For*N. cati*, efficacy was determined using a modified formula (Hampel et al., 2018): 


$$\left[\left(\text{B - P}\right)\text{/}\left(\text{B}\right)\right]\text{x 100}$$ (2)

where B is the pre-treatment mean number of live mites, and P is the post-treatment mean for either the investigational product or positive control group at each evaluation point.

### Statistical analysis

Parasite counts and clinical scores were analyzed using non-parametric methods. The Mann–Whitney U test (Wilcoxon rank-sum) was applied for comparisons between two independent groups. For comparisons involving three groups, the Kruskal–Wallis test was used, followed by pairwise post hoc analyses. Statistical significance was set at*p*≤ 0.05. Analyses were conducted using IBM SPSS Statistics, version 29.0.1.0 (171).

## Results

### Investigation of adverse events

No adverse events associated with the administration of the investigational product containing fipronil, moxidectin and praziquantel or any of the positive control treatments were observed in any of the three studies.

### Efficacy against*Otodectes cynotis*

Pre-treatment comparisons of mean mite counts among the three experimental groups revealed no statistically significant differences (*P* = 0.872). No significant differences in mean mite counts were observed between the groups treated with the investigational product, positive control, or negative control at baseline or at assessments conducted 7 and 2 days prior to treatment (*P* > 0.05) (Table 1). In all post-treatment evaluations (days +3, +7, +14, +21, and +28), animals in both treated groups were negative for mite presence. In contrast, animals in the negative control group remained positive throughout the post-treatment period, with mite counts ranging from 2.5 to 15.5 mites (average of both ears) (Table 1). Post-treatment comparisons among the three groups indicated that the negative control group differed significantly from both treated groups (*P* = 0.000). No significant difference was observed between the group treated with 6% selamectin and the group treated with the investigational product (*P* = 1.000) (Table 1). Both treatments achieved 100% efficacy by day +3, and treated animals remained mite-free through all subsequent evaluations.

Otoscopy findings corroborated these results, with no live mites detected in either treated group at any post-treatment assessment point (days 3, 7, 14, 21, and 28) (*P* = 0.000) (Table 1). Similarly, no statistically significant differences were found between the positive control and investigational product groups, further confirming equivalent efficacy. Following ear canal lavage, no live mites were recovered from cats in either treated group (Table 2), confirming 100% efficacy under controlled environmental conditions.

**Table 2:** Mean mite counts of *Otodectes cynotis* in cat ear canals on day 28 post-treatment, following ear canal washing.

**Group**	**Mite count after ear canal flushing**
**Negative Control Group**	
**Mean (sd)**	10.6 (2.41)
**Positive Control Group**	
**Mean (sd)**	0
**Efficacy (%)**	100.0
**Treated Group**	
**Mean (sd)**	0
**Efficacy (%)**	100.0
***P-value***	0.0015

Negative control group = cats without treatment; Positive control = cats that have received topical 6% selamectin; Treated group = cats that have received topical administration of the investigational product; sd = standard derivation; *P* value comparing mite counts among groups.

### Efficacy against*Notoedres cati*

Statistical analysis comparing pre-treatment and post-treatment mean mite counts (days 3, 7, 14, 21, and 28) demonstrated a reduction mean counting from 93.83 to 1.17 and from 155.50 to 9.50 in both the group treated with the investigational product and the Positive Control Group (10% imidacloprid + 1% moxidectin) respectively (*P* < 0.0001) (Table 3). Mites were absent from both groups after the day +7 evaluation, achieving 100% efficacy. Similarly, comparisons of pre-treatment injury scores (day -2) with post-treatment results showed improvements from score 3 to 1 in both treatment groups (*P* = 0.0277) (Table 4). Treatments effectively resolved lesions observed at baseline, achieving maximum (100%) efficacy in naturally infested, indoor-housed animals. No statistically significant differences were noted between the treated and positive control groups in injury scores at any post-treatment time point (days 3, 7, 14, 21, and 28) (*P* = 0.6310), indicating comparable effectiveness between the two treatments (Table 4).

**Table 3:** Mean count of *Notoedres cati* among groups on different evaluation days.

**Group**	**Experimental day**					
	-2	3	7	14	21	28
**Positive Control Group**						
Positive animals	6	4	0.0	0.0	0.0	0.0
Mite score (min – max)	29 - 176	0 - 25	0.0	0.0	0.0	0.0
Mean (sd)	155.50 (109.1)	9.50 (20.4)	0.0	0.0	0.0	0.0
**Efficacy (%)**	**- - -**	**93.9**	**100.0**	**100.0**	**100.0**	**100.0**
P value intra group	- - -	<0.0001	<0.0001	<0.0001	<0.0001	<0.0001

**Treated Group**						
Positive animals	6	2	0.0	0.0	0.0	0.0
Mite score (min – max)	27-54	0-2	0.0	0.0	0.0	0.0
Mean (sd)	93.83 (24.3)	1.17 (1.8)	0.0	0.0	0.0	0.0
**Efficacy (%)**	**- - -**	**100.0**	**100.0**	**100.0**	**100.0**	**100.0**
*P* value intra group	- - -	<0.0001	<0.0001	<0.0001	<0.0001	<0.0001
						
*P* - value inter group	0.423	0.471	- - -	- - -	- - -	- - -

Positive control = cats that have received topical Imidacloprid 10% and Moxidectin 1%; Treated Group = cats that have received topical administration of the investigational product; min = minimum; max = maximum; sd = standard derivation; *P* value comparing mite counts between the experimental groups (Positive Control Group versus Treated Group) and among the groups.

**Table 4:** Group severity scores of skin lesions observed in cats naturally infested with *Notoedres cati*, in treated and untreated groups.

**Group**	**Experimental day**					
	-2	3	7	14	21	28
**Positive Control Group**						
Mean (sd)	3.0 (0.6)	2.50 (0.8)	2.0 (0.6)	1.3 (0.8)	1.2 (0.4)	1.17 (0.4)
*P-* value intragroup	- - -	0.109	0.043	0.027	0.027	0.027
						
**Treated Group**						
Mean (sd)	3.0 (0.63)	2.8 (0.8)	2.2 (0.4)	1.5 (0.6)	1.17 (0.4)	1.0 (0.0)
*P- value* intragroup	- - -	0.3173	0.0431	0.0277	0.0277	0.0277
						
*P- value* intergroup	1	0.423	0.689	0.4712	1.0000	0.6310	

Score: 0 - No skin lesions, no alopecia, no itching; 1 - Mild skin lesions, mild alopecia, occasional itching; 2 - Moderate skin lesions, moderate alopecia, occasional itching; 3 - Severe skin lesions, large areas of alopecia, presence of crusts, intense itching, ulcerated lesions; Positive Control Group, treated with 10% imidacloprid + 1% moxidectin; Treated Group, treated with the investigational product; sd = standard derivation; *P* value comparing mite counts intergroup and intragroup.

### Efficacy against*Felicola subrostratus*

Post-treatment evaluations revealed a significant difference between the control group and the group treated with the investigational product on days 3, 7, 14, 21, and 28 (*P* = 0.0017). No lice were detected in the treated group after day +3, achieving 100% efficacy when evaluated in naturally infested cats maintained under indoor conditions (Table 5).

**Table 5:** Mean count of *Felicola subrostratus* among groups on different evaluation days.

**Group**	**Experimental day**						
	-7	-2	3	7	14	21	28
**Negative Control Group**							
Positive animals	7	7	7	7	7	7	7
Lice score (min – max)	218-412	281-374	236-363	156-411	240-394	269-344	278-442
Mean (sd)	300.4 (58.9)	330.0 (34.9)	303.3 (43.7)	283.6 (78.4)	313.1 (52.5)	320.9 (27.6)	338.0 (56.7)

**Treated Group**							
Positive animals	7	7	0	0	0	0	0
Lice score (min - max)	261-349	318-421	0	0	0	0	0
Mean (sd)	311.7 (35.4)	365.4 (31.9)	0	0	0	0	0
**Efficacy (%)**	**- - -**		**100.0**	**100.0**	**100.0**	**100.0**	**100.0**
							
*P* - value	0.6718	0.0707	0.0017	0.0017	0.0017	0.0017	0.0017

Negative control group = cats without treatment; Treated group = cats that have received topical administration of the investigational product; sd = standard derivation; min = minimum; max = maximum; *P* value comparing mite counts among groups*.*

## Discussion

This is the first study to evaluate a single topical formulation combining fipronil, moxidectin, and praziquantel for its efficacy against *O. cynotis*, *N. cati*, and *F. subrostratus*in domestic cats. The formulation demonstrated high efficacy, achieving 100% effectiveness against*O. cynotis* and *F. subrostratus* by Day 3 post-treatment, and against*N. cati*by Day 7.

The immediate and sustained efficacy of ectoparasiticides is critical in field settings, where cats may experience repeated exposure to parasites, adverse reactions due to high parasite loads, or face challenges related to inadequate control and emerging resistance. The investigational product, which showed mortality against other species (Campos et al., 2025) demonstrated 100% efficacy for 28 days following a single application, indicating its ability to eliminate both adult parasites and developing stages that would later emerge from eggs present on the host’s haircoat or skin. This suggests that the formulation was effective against immature stages hatching from residual eggs, thereby ensuring complete parasite elimination during the study period. However, no reinfestation or challenge studies were conducted, and therefore, it is not possible to state that the product provides protective residual efficacy against new infestations beyond this period. The formulation effectively resolved clinical signs of otodectic mange and eliminated mite counts following a single administration. Both the test and positive control groups achieved 100% acaricidal efficacy against*O. cynotis*, indicating that the investigational formulation is highly effective in treating naturally infested cats. The observed efficacy aligns with previous studies demonstrating the acaricidal activity of fipronil and moxidectin against*O. cynotis*(Yang & Huang, 2016; Balandina et al., 2017). Moxidectin, a macrocyclic lactone with systemic distribution, likely targets mites within the ear canal during blood or tissue feeding (Prichard et al., 2012). Fipronil, by contrast, exerts its effect through topical contact, disrupting mite populations on the skin surface and during their migration into the ear canals (Strugaru et al., 2018). The lipophilicity of moxidectin allows it to reach high concentrations in the skin and associated tissues (Knaus et al., 2014), further enhancing its local antiparasitic activity.

The investigational formulation achieved complete elimination of *O. cynotis* after 3 days of administration, an outcome that compares favorably to previous reports in which treatments such as ivermectin, selamectin, and doramectin required longer durations and multiple doses to attain partial or complete efficacy (Yang & Huang, 2016). Reported efficacy rates in these studies ranged from 50% to 90% over treatment periods of 14–28 days. In contrast, the present formulation (12.5% fipronil, 0.83% moxidectin, 8.3% praziquantel) achieved 100% efficacy after a single dose after three days, highlighting its superior performance and clinical relevance.

The topical formulation administered once at the recommended dosage to cats naturally infested with *N. cati*, achieved 100% efficacy, as determined by mite counts on Day 3 post-treatment. In addition to its acaricidal effect, the investigational formulation (12.5% fipronil, 0.83% moxidectin, 8.3% praziquantel) also resulted in marked clinical improvement in signs of notoedric mange. Statistical analysis of mite counts revealed no significant differences between the treated group and the positive control group, indicating that the performance of the investigational product was comparable to that of the commercial reference product. Treatment of feline notoedric mange commonly involves various topical or injectable agents, with current protocols frequently incorporating macrocyclic lactones in diverse formulations (Lanusse et al., 2018). The acaricidal activity observed in the present study is likely attributable to moxidectin (Balandina et al., 2017), although fipronil, which is approved for aiding in the control of mange caused by*Sarcoptidae*mites, may also contribute to efficacy by limiting infestations (Little & Cortinas, 2023). While fipronil alone is not recommended as a monotherapy for notoedric mange, its inclusion in combination formulations may provide a valuable adjunct to treatment when appropriately paired with other active ingredients. A similar combination product containing fipronil, (*S*)-methoprene, eprinomectin, and praziquantel (Broadline®, Merial) demonstrated acaricidal efficacy against*N. cati*within 14 days (Knaus et al., 2014), whereas the formulation evaluated in the present study achieved the same level of efficacy within only three days, representing a more rapid onset of action. For *F. subrostratus*, significant differences were observed between the negative control group and the group treated with the investigational product as early as Day 3, with 100% efficacy maintained throughout the study period. Few studies have evaluated the efficacy of insecticides, particularly spot-on or spray-based formulations containing fipronil, against *F. subrostratus*in cats (Pollmeier et al., 2004). However, macrocyclic lactones have shown effectiveness in this context. A formulation combining esafoxolaner, eprinomectin, and praziquantel (NexGard Combo®) demonstrated efficacy against *F. subrostratus* by Day 14 post-treatment (Mihalca et al., 2022), whereas the formulation tested in the present study achieved full efficacy within three days.

Taken together, the findings of this study confirm that the investigational formulation, applied topically in a single dose, is effective in treating natural infestations of*O. cynotis*,*N. cati*, and*F. subrostratus*in domestic cats. The formulation achieved 100% efficacy up to 28 days post-treatment, with maximal efficacy observed by Day 3 for*O. cynotis*and*F. subrostratus*, and by Day 7 for*N. cati*, supporting its use as an effective therapeutic option for feline ectoparasitic infestations.

## Conclusion

The findings from this study demonstrate that the investigational product containing 12.5% fipronil, 0.83% moxidectin, 8.3% praziquantel, administered topically in a single dose, is effective against *O. cynotis*, *N. cati*, and *F. subrostratus* in naturally infested cats.

## Data Availability

The datasets generated and/or analyzed during the current study are available from the corresponding author on reasonable request.

## References

[B001] Arisov MV, Indyuhova EN, Arisova GB (2018). Pharmacokinetics of combination antiparasitic drug preparation for dogs and cats in the form of spot-on solution. J Adv Vet Anim Res.

[B002] Balandina VN, Kryuchkova EN, Arisov MV (2017). Efficiency of moxidectin at Otodectes cynotis and Notoedres cati infections of cats.

[B003] Becskei C, Reinemeyer C, King VL, Lin D, Myers MR, Vatta AF (2017). Efficacy of a new spot-on formulation of selamectin plus sarolaner in the treatment of Otodectes cynotis in cats. Vet Parasitol.

[B004] Campos DR, Marré EO, Cruz BC, Cruz GAM, Michels MG, Gatto IRH (2025). Efficacy of a topical formulation combining fipronil, moxidectin, and praziquantel (Banni3) in controlling flea infestation in cats. Front Vet Sci.

[B005] Campos M, Freitas ND, Gomes DE (2019). Otodecic mange – a review. Sci Mag.

[B006] Chai JY (2013). Praziquantel treatment in trematode and cestode infections: an update. Infect Chemother.

[B007] European Agency for the Evaluation of Medicinal Products (2000). VICH GL9: good clinical practice.

[B008] Figueiredo MAP, Manrique WG, War RMSNC (2013). Felicola subrostratus parasitizing domestic cats in São Luís, Maranhão, Brazil: case report. Biotemas.

[B009] Gant DB, Chalmers AE, Wolff MA, Hoffman HB, Bushey DF (1998). Fipronil: action at the GABA receptor. Rev Toxicol.

[B010] Hampel V, Knaus M, Schäfer J, Beugnet F, Rehbein S (2018). Treatment of canine sarcoptic mange with afoxolaner (NexGard®) and afoxolaner plus milbemycin oxime (NexGard Spectra®) chewable tablets: efficacy under field conditions in Portugal and Germany. Parasite.

[B011] Hellmann K, Petry G, Capári B, Cvejic D, Krämer F (2013). Treatment of naturally Notoedres cati-infested cats with a combination of imidacloprid 10%/moxidectin 1% spot-on (Advocate®/Advantage® Multi, Bayer). Parasitol Res.

[B012] Knaus M, Capári B, Visser M (2014). Therapeutic efficacy of Broadline® against notoedric mange in cats. Parasitol Res.

[B013] Lanusse CE, Imperiale FA, Lifschitz AL, Papich MG, Riviere JE (2018). Veterinary Pharmacology & Therapeutics.

[B014] Little SE, Cortinas R, Sykes JE (2023). Greene’s infectious diseases of the dog and cat..

[B015] Lucas R, Feitosa FLF (2008). Veterinary semiology - the art of diagnosis.

[B016] Mihalca AD, Deak G, Panait LC, Rabei S, Beugnet F (2022). Efficacy of a topical formulation containing esafoxolaner, eprinomectin, and praziquantel (NexGard Combo®) against natural infestations with the cat louse, Felicola subrostratus under field conditions. Parasite.

[B017] Miller WH, Griffin CE, Campbell KL, Miller WH, Griffin CE, Campbell KL (2012). Muller and Kirk’s small animal dermatology.

[B018] Pollmeier M, Pengo G, Longo M, Jeannin P (2004). Effective treatment and control of biting lice, Felicola subrostratus (Nitzsch in Burmeister, 1838), on cats using fipronil formulations. Vet Parasitol.

[B019] Premaalatha B, Fazly A, Faizah HM, Farah HMT (2023). Ectoparasite infestation on domestic cats in Ipoh, Perak. Malays J Vet Res.

[B020] Prichard R, Ménez C, Lespine A (2012). Moxidectin and the avermectins: consanguinity but not identity. Int J Parasitol Drugs Drug Resist.

[B021] Rataj AV, Posedi J, Bidovec A (2004). Ectoparasites: *otodectes cynotis, Felicola subrostratus* and *Notoedres cati* in the ear of cats. Slov Vet Res.

[B022] Santos TC, Silva BRF, Reggiani DG, Campos ML, Roldan JAM, Onofrio VC, Moraes-Filho J (2019). Escabiose felina no gato errante – Relato de caso. Braz J Develop.

[B023] Schraven AL, Stannard HJ, Old JM (2021). A systematic review of moxidectin as a treatment for parasitic infections in mammalian species. Parasitol Res.

[B024] Sivén M, Savolainen S, Räntilä S, Männikkö S, Vainionpää M, Airaksinen S (2017). Difficulties in administration of oral medication formulations to pet cats: an e-survey of cat owners. Vet Rec.

[B025] Stanneck D, Kruedewagen EM, Fourie JJ, Horak IG, Davis W, Krieger KJ (2012). Efficacy of an imidacloprid/flumethrin collar against fleas, ticks, mites and lice on dogs. Parasit Vectors.

[B026] Strugaru SA, Nicoara M, Robea MA, Plavan G, Ciobica A (2018). Fipronil: mechanisms of action on various organisms and future relevance for animal models studies. J Surv Fish Sci.

[B027] Taenzler J, De Vos C, Roepke RKA, Frénais R, Heckeroth AR (2017). Efficacy of fluralaner against *Otodectes cynotis* infestations in dogs and cats. Parasit Vectors.

[B028] Taylor S, St. Denis K, Collins S, Dowgray N, Ellis SL, Heath S (2022). 2022 ISFM/AAFP Cat Friendly Veterinary Environment Guidelines. J Feline Med Surg.

[B029] Thomas JE, Staubus L, Goolsby JL, Reichard MV (2016). Ectoparasites of free-roaming domestic cats in the central United States. Vet Parasitol.

[B030] Yang C, Huang HP (2016). Evidence-based veterinary dermatology: a review of published studies of treatments for Otodectes cynotis (ear mite) infestation in cats. Vet Dermatol.

